# On the First Transmission of Cow-Pox Virus to America

**Published:** 1817-05

**Authors:** T. J. Pettigrew


*For the London Medical and Physical Journal.*

### *On the first Transmission of Cow-pox Virus to America;* by T. J. PETTIGREW, Esq.

IN the last Number of your Journal, a correspondent, under the signature of Georgiensis, expresses his surprise to find it stated, in my Memoirs of the Life and Writings of the late Dr. Lettsom, that the vaccine lymph was first sent across the Atlantic by Dr. L., and consigned to the care of his friend Dr. Waterhouse, of Cambridge, Massachusetts, from whence it *spread* through the United States. This is said to be untrue: that "vaccine lymph had been previously sent by Dr. George Pearson to Dr. Chichester, now a resident physician at Bath, but at that time in very extensive practice in Charleston in South Carolina." Reference is also given to the Phil. Mag. vol. xvi. p. 252, for the particulars of the vaccination of *one* individual, in the winter of 1799, by Dr. C.

Nothing can be further from my wish than to attribute that to any individual which does not appear to be justly his due; and, had I not been informed by the HIGHEST authority on this subject, that Dr. Lettsom had been the first to transmit to America this inestimable treasure, I certainly should not have ventured to state it in my publication. The notice of your correspondent has, however, induced me to examine a number of letters written by Dr. Waterhouse to Dr. Lettsom, and the notes of letters transmitted by Dr. Lettsom to Dr. Waterhouse, to be convinced of the truth or error of the statement I have made. From this examination, I am free to confess, that Dr. L. does not appear to have *first* sent the vaccine lymph across the Atlantic.

I subjoin

I subjoin extracts from these letters, that your readers may draw their own conclusion with respect to dates. I cannot, however, forbear expressing some degree of surprise, that the practice of vaccination was not followed up by Dr. Chichester; for it is rather singular, that no mention of his name occurs but in the statement contained in the Phil. Mag., unless it be in the writings of Dr. George Pearson, which I happen not to be in possession of, and to which, at present, it is not in my power to refer. Dr. Waterhouse, on the contrary, is frequently alluded to by various writers. He was the active promoter of the practice in America—the person to whom the members of the government applied for lymph, and for directions for its use; and to whom his professional brethren looked for information on this subject. He seems, also, from the following extracts, justly to feel the responsibility of his situation on the occasion. If, therefore, Dr. W. was not the first (which I confess appears to me doubtful) who vaccinated in America, it is but due to admit that it was by him that the practice *spread* through, and was finally established in, the United States.

*Dr. W. to Dr. L.*

*Cambridge; April* 10, 1799.—"I received with great satisfaction your letter of the 24th Nov. with Dr. Jenner's and Dr. Pearson's publications on a new, curious, and extremely important disease. I directly threw an account of it into the newspapers, a copy of which I here enclose. I should be highly gratified by more information respecting this epizootic disorder, and of further trials on the human kind. As such a distemper has never been heard of in this country, it excites the public curiosity as much as any thing that has occurred in the medical line since my remembrance."

*Nov.* 14, 1799.—"I here enclose a letter for Dr. Woodville, as it is making the same request I did to you respecting some cow-pox matter.* I send it opened, and wish you would be so kind as to put a wafer in it, and suffer the penny post to convey it to him. The curiosity, nay anxiety, of the public, especially of parents, on this subject, is very considerable. We, indeed, feel anxious ourselves, as four of our six children have never been inoculated."

*Nov.* 13, 1800.—"I am in no small tribulation for want of the vaccine matter. I introduced it into this country, but

* From this passage it seems probable that Dr. W. had previously solicited vaccine lymph from Dr. L. No letter, however, containing a request of this kind is preserved.—P.

some how or other it has depreciated in my hands. It fails in more than half I have inoculated for several weeks past. I never received any but from Dr. Haygarth, which was last June.

"I have never been able to procure Woodville's last publication on the Cow-pox. Are there any good practical treatises recently published on this subject? As I was the first who introduced it here, I am applied to from all quarters, but am chagrined almost to sickness because I have no confidence in the matter I possess. The vaccine poison has become milder by passing through a number of the human species, or else the cold weather has deprived it of half its venom. As soon as I receive fresh matter from England, I will directly inoculate a cow, by way of obtaining active matter from the fountain head.

"I have had several instances in the cow-pox, where the symptoms came on pretty violently in twenty-four hours. In many instances I am puzzled to know whether the patient has really gone through the disease so as to secure him from further infection. My situation is peculiarly perplexing, for, should any unfortunate case occur under any practitioner, I shall bear the blame of it. I have diffused the matter all over the country, and am conscious that it has degenerated and become spurious. Applications by letter and otherwise crowd upon me every hour, and almost every minute, to solve doubts, give directions, and console disappointments; and I have no person to apply to myself for the information which I feel I myself stand in need of. I have Jenner's Work (1st and 2d part), Woodville's first publication, and Pearson's first pamphlet, and the 2d vol. of the Medical and Physical Journal; and could wish that Mr. Mawman would send me any thing and every thing that has, or may, come out in the course of the winter, *which you can recommend.*"

*Dec.* 13, 1800.—"As I know not Dr. Jenner's address, I have enclosed a letter, which I would thank you to forward to him as soon as possible. I have written to him on a subject in which I am deeply interested—I mean the cow-pox. You already know, perhaps, that I introduced that distemper here, and led the way in its inoculation, and that very much to my advantage; but it has lately worked very perversely, and occasioned me much perplexity. Since the cold raw weather of November came in, the *matter* has *deteriorated* in my hands, and in the hands of every one else, so that almost all the cases that have lately occurred have proved *spurious*; and, unless I can obtain a fresh supply of the vaccine matter early in the spring, the inoculation for it will sink sink into disrepute, and I myself come in for a large portion of the disgrace.

"Judge of my anxiety when I say that I am conscious that more than an hundred practitioners in different parts of New England are at this time inoculating with *spurious* matter, while the small-pox is pervading one of our sea ports, and every unfortunate case will be traced up to me, the originator of the practice in America. I am the only person who ever succeeded in obtaining efficient matter from England, which I had from Dr. Haygarth, and the activity of this matter *seems worn out by passing through a number of the human species;* and, unless I obtain a fresh supply from England, the business which promised so fair here will stagnate. I have, therefore, written to Dr. Jenner for his advice and assistance. So I have to Dr. Pearson, and hope, by the return of the Galen, or by the Minerva, to get a supply of the matter I stand so much in need of. I have just received an order from the War-Department to supply the military surgeons with the vaccine matter, and directions for inoculating the different corps of artillerists and engineers stationed in the various parts of New England."

*Dr. L. to Dr. W.*

*London; March* 28, 1800.—" Sent him fresh cow-pox matter."

*Dec.* 24.—" Sent him vaccine matter in a glass bottle from the Vaccine Institution, as well as two planes of glasses, one from Dr. Woodville, and the other from Mr. Johnson. Explained Dr. Woodville's, Dr. Pearson's, and Dr. Jenner's opinions respecting the wearing out of the effect of the matter."

*Feb.* 19, 1801.—" Sent him vaccine matter in a glass vessel from myself, with a letter and vaccine matter from Dr. Pearson."

*Bolt-court, Fleet-street;*
*April* 16, 1817.

